# Patient-to-Patient Transmission of Hepatitis C at Iranian Thalassemia Centers Shown by Genetic Characterization of Viral Strains

**DOI:** 10.5812/hepatmon.7699

**Published:** 2013-01-23

**Authors:** Katayoun Samimi-Rad, Freshteh Asgari, Mohsen Nasiritoosi, Abdoulreza Esteghamati, Azar Azarkeyvan, Seyedeh Masoomeh Eslami, Farhad Zamani, Lars Magnius, Seyed Moayed Alavian, Heléne Norder

**Affiliations:** 1 Department of Virology, School of Public Health, Tehran University of Medical Sciences, Tehran, IR Iran; 2 Center for Disease Control, Deputy of Health, Ministry of Health and Medical Education, Tehran, IR Iran; 3 Department of Internal Medicine, Gastroenterology and Hepatology Section, Tehran University of Medical Sciences, Tehran, IR Iran; 4 Department of Pediatric, Tehran University of Medical Sciences, , Tehran, IR Iran; 5 Iranian Blood Transfusion Organization (IBTO), Thalassemia Center,, Tehran, IR Iran; 6 Gastrointestinal and Liver Disease Research Center, Firoozgar Hospital, Tehran University of Medical Sciences, , Tehran, IR Iran; 7 Department of Microbiology, Tumor and Cell Biology (MTC), Karolinska Institute, Solna, Sweden; 8 Research Center for Gastroenterology and Liver Disease, Baqiatallah University of Medical Sciences, , Tehran, IR Iran; 9 Department of Infectious Diseases, University of Gothenburg, Gothenburg, Sweden

**Keywords:** Hepatitis C, Thalassemia, Iran

## Abstract

**Background:**

Hepatitis C is prevalent among thalassemia patients in Iran. It is mainly transfusion mediated, in particular among patients treated before 1996 when blood screening was introduced.

**Objectives:**

The current study aimed to investigate why patients still seroconvert to anti-HCV in Iranian thalassemia centers.

**Patients and Methods:**

During 2006-2007 sera were sampled from 217 anti-HCV positive thalassemia patients at nine thalassemia centers in Tehran and Amol city, where 34 (16%) patients had been infected after 1996. The HCV subtype could be determined by sequencing and phylogenetic analysis of partial NS5B and/or 5׳NCR-core region in 130 strains.

**Results:**

1a (53%) was predominant followed by 3a (30%), 1b (15%), and one strain each of 2k, 3k and 4a. Phylogenetic analysis revealed 19 clades with up to five strains diverging with less than six nucleotides from each other within subtypes 1a and 3a. Strains in seven clades were from nine patients infected between 1999 and 2005 and similar to strains from eight patients infected before 1996, indicating ongoing transmission at the centers. Further epidemiological investigation revealed that 28 patients infected with strains within the same clade had frequently been transfused at the same shift sitting on the same bed. An additional eight patients with related strains had frequently been transfused simultaneously in the same room.

**Conclusions:**

The results suggest nosocomial transmission at these thalassemia centers both before and after the introduction of blood screening. Further training of staff and strict adherence to preventive measures are thus essential to reduce the incidence of new HCV infections.

## 1. Background

Thalassemia major and thalassemia intermedia are both common transfusion dependent anemias in Iran. There are around 18000 known thalassemia patients in the country (1). Reports from different regions of Iran estimate that 18% of the thalassemia patients are positive for anti-HCV (1-3), which is far higher than the positivity rate of 0.5% found in the general Iranian population (4). Most of the thalassemia patients live in Tehran (n = 2,850) and Mazandaran (n = 2,880) provinces. Transfusion of unscreened blood was previously the main risk factor for HCV infection in thalassemia patients, but this risk was reduced after 1996 when screening for anti-HCV was introduced at all blood banks in Iran (5, 6). In 2005 the Ministry of Health and Medical education implemented a national plan to decrease the rate of infection in thalassemia patients by offering free anti-HCV testing and anti-viral treatment to those found positive. This supplementary strategy has decreased the number of newly infected, although new infections still occur at low rate. This may be due to inaccurate blood screening or by transfusion of blood collected from hepatitis C infected donors during the window period before anti-HCV appears. Other nosocomial exposures may also play a role in HCV transmissions. Sequence analysis of the infecting HCV strains is now a powerful tool to trace infectious sources and thereby also transmission routes (6).

## 2. Objectives

The current study aimed to characterize HCV strains from patients at thalassemia centers in Tehran in Tehran province and in Amol City in Mazandaran province to investigate possible nosocomial transmission at these centers.

## 3. Patients and Methods

### 3.1. Study Population

Two hundred seventeen thalassemia patients positive for anti-HCV were included in the study. Out of these, 112 patients were from seven thalassemia centers in Tehran, Zafar adult thalassemia clinic, Childrens׳ medical center , Sodeh clinic, Aliasghar Hospital, Mofid Hospital, Special Medical center, Boali Hospital and from Shahid Bahonar hospital in Karaj City. The other 105 patients were from Amol thalassemia center at Imam Reza hospital in northern Iran. All staff members in Amol and Zafar adult thalassemia centers were anti-HCV negative. Table 1 indicates the patients` information, age and gender. The patients received blood transfusions, every two to four weeks and had been regularly tested for anti-HCV every six months or once a year at least since 2001. Information was obtained by a questionnaire on date of birth, gender, age at first transfusion, duration of transfusion therapy, number of transfusions received until the time of sampling, risk factors, and date of admission to the thalassemia center. Retrospectively, further interviews were conducted in 2008 on 36 patients found infected with similar HCV strains. The purpose of the study was explained to the patients or to the parents of the children and informed consent was obtained before sampling. This study was conducted in compliance with the World Medical Association Declaration of Helsinki and was approved by Tehran University of Medical Sciences Ethics Committee. All anti-HCV positive patients diagnosed as thalassemic were included in the study except those also seropositive for hepatitis B virus (HBV) or immunodeficiency virus (HIV). All the patients fromTehran and Amol were bled during 2006 and 2007, respectively.

**Table 1:** Characteristics of 217 Thalassemia Patients Transfused in Tehran and Amol and PCR and Sequencing Results of Their HCV Strains

Region	No.	Males/Females	Age, y, (min-max)	Mean ± SD	HCV PCR Positive		No. of Sequenced Strains	
					NS5B, No. (%)	Core, No. (%)	NS5B, No. (%)	Core, No. (%)
**Tehran**	112	56/56	12-47	25.2 ± 7.0	60 (54)	13 (24) a	58 (97)	11 (85) a
**Amol**	105	56/49	11-63	21.5 ± 7.4	58 (55)	3 (6)	58 (100)	3 (100)
**Total**	217	112/105	11-63	23.4 ± 7.4	118 (54)	16 (16)	116 (98)	14 (88)

^a^Including two samples which could be amplified but not sequenced based on NS5B region

### 3.2. RNA Extraction and PCR

100 µl plasma was added to 500 µl lysis buffers (0.5% SDS and 10mM EDTA) and 500 µl water saturated phenol. RNA was precipitated with isopropanol. The pellet was washed with 70% ethanol and dissolved in 20 µl distilled water. cDNA synthesis and semi-nested PCR were performed with primers hep101, hep102 and hep105 as previously described (7, 8) to give a 380-bp product between positions 8258 and 8687 of NS5B. The 5'UTR- core region was also amplified for several strains using the primers 186/NCR3 and 186/univ-1 (8).

### 3.3. Sequencing and Phylogenetic Analysis

**Figure 1:**
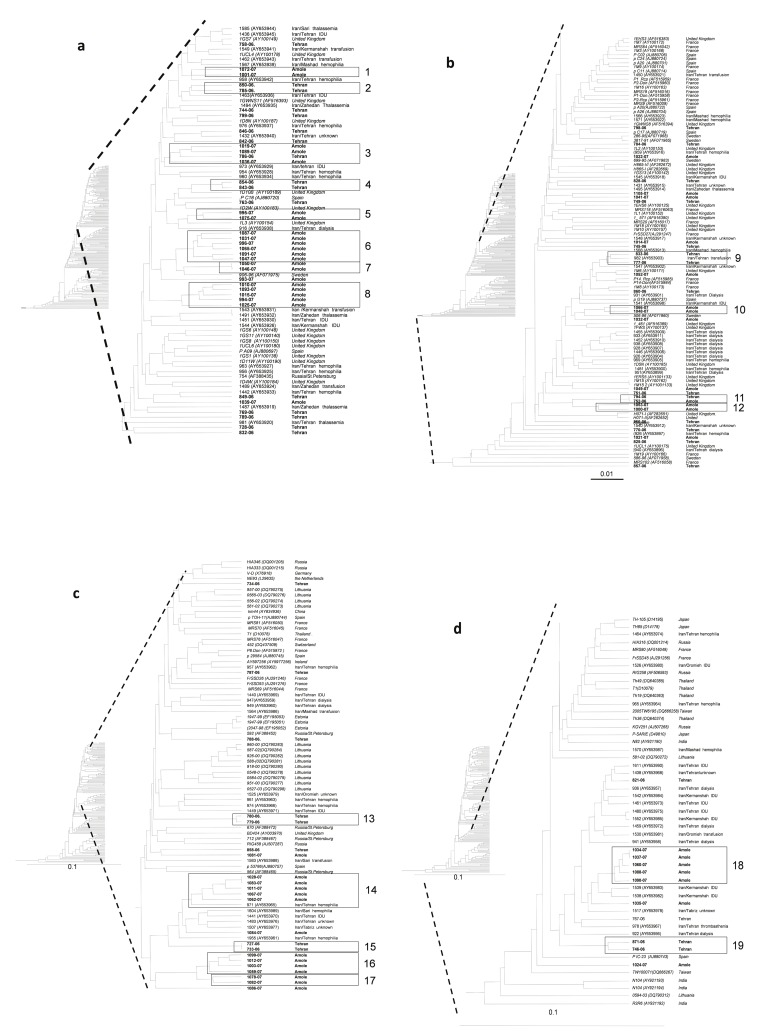
UPGMA dendrogram based on 325 nucleotide of the NS5B region of 379 HCV strains The thalassemia strains of this study are showed bold and other strains in italic. The designations and origin of the strains obtained from GeneBank, are given at the nodes. The F84 model of nucleotide substitution was used to estimate genetic distances with a single category of substitution rates and gamma distributed rates across sites with alpha 0.74, empirical base frequencies, and transition/transversion ratios of 5.9 for genotype 1, 2.88 for genotype 2, 3.68 for genotype 3 and 2.0 for genotype 4. The clades with their designations are marked in the phylogenetic tree .a and b: part of the phylogenetic tree showing the clusters formed by the subtype 1a sequences. c and d: part of the phylogenetic tree with the branches formed by subtype 3a.

The products obtained from the NS5B and 5'UTR-core regions were purified using GFX PCR DNA and Gel band purification kit (GE Healthcare, Buckinghamsure, UK). The sequencing reaction was carried out with the ABI PRISM TM Big Dye TM Terminator Cycle Sequencing Reaction Kit (Applied Biosystem, Foster city, CA, USA, version 3.1) using purified PCR products as templates. PCR products amplified within the NS5B region were sequenced with hep105, and those amplified within the 5'UTR-core region were sequenced with primers univ-1 and 186. The obtained sequences were aligned with 347 sequences of the corresponding region retrieved from Gen Bank (see accession numbers in Figure 1). The genetic distances between the aligned sequences were calculated using the F84 model in DNADIST in the Phylip program package version 3.66c. Phylogenetic trees were constructed using the UPGMA algorithm in the program NEIGHBOUR in the Phylip program package. The sequences obtained in this work are deposited in GenBank with accession numbers KC118130- KC118333.

### 3.4. Statistical Analysis 

Statistical analysis was performed with Fisher’s test. P < 0.05 was considered statistically significant.

## 4. Results

Most of the patients had been hepatitis C infected before 1996, when testing for anti-HCV and blood screening was introduced in Iran. However, 34 (16 %) patients had been infected after 1996, most of them, 26 (76 %), were infected after 2000 (Table 2). Fourteen of these patients were from Tehran and 20 from Amol. The thalassemia centers have separate transfusion rooms for men and women, and several patients are simultaneously transfused at the same shift in the same room. HCV RNA was detected in sera from 132 (61 %) patients. The subtype could be determined in 130 of them, 69 from Tehran and 61 from Amol (Table 1). The NS5B region was amplified and sequenced for 116 strains and the core region only for 14 strains (Table 1). The most common subtype found from the Tehran centers was 1a in 34 (49%) patients followed by 1b in 17 (25%) and 3a in 15 (22 %). Strains of subtypes 2k, 3k, and 4a were found infecting one patient each (Table 3). Patients at the center in Amol were also mainly infected by 1a strains (n = 35; 57 %), whereas 1b was found only in two, which was significantly less thanthat of Tehran (P < 0.001). Subtype 3a was somewhat more frequent in Amol compared to Tehran, 39 % versus 22% (Table 3). In the NS5B region, there were significantly more similar 1a strains, diverging with less than six nucleotides from each other, in Amol center than in Tehran centers, 24/35; 69 % versus 6/34; 18 % (P ≤ 0.001). Phylogenetic analysis of the NS5B region revealed high divergence between the strains. However, strains from 57 patients diverged with less than six nucleotides from strains of at least one other patient, and formed 19 clades (Figure 1a-d). Seventeen (89 %) of these clades were formed by strains from patients of the same sex (Table 4). The majority of the patients with similar strains, 47 (82 %), had been infected before 1996. The strains from the 10 patients infected after 1996 were found in eight of the 19 clades. They did not form separate clades, and were mostly similar to strains from patients infected before 1996 (Table 4). Clades one to 12 were formed by 33 (52.3%) of the 65 subtype 1a strains in this study (Figure 1a-b, Table 4). Four of these (clades 2, 4, 9, and 11) were formed exclusively by strains from Tehran patients. The patients infected with strains in three of these clades (4, 9 and 11) had frequently been transfused in the same room at the same shift (Table 4).

 Strain 752 in clade 11 was from a patient infected in 1999 and identical to strain 794 from a patient infected before 1996. Seven clades were formed by strains only from Amol (clades 1, 5, 6, 7, 8, 10 and 12; Figure 1a-b). An additional clade, 3, was formed by strains mainly from Amol, but also with one strain from Tehran. Strains in six of these 1a clades were from 16 patients (eight pairs) who had been transfused simultaneously sitting on the same bed at the center in Amol (Table 4). Two of the eight pairs were formed by one patient each infected before and after 1996. Seven clades were formed by 22 of the 37 (59%) subtype 3a strains in the current study study (Figure 1c-d). Three clades were formed by strains from Tehran (clades 13, 15 and 19) and four by strains from Amol (clades 14, 16, 17 and 18; Table 4; Figure 1 c-d). There were significantly more closely related 3a strains among patients from Amol as compared to those of Tehran, 73% versus 40%, respectively (P < 0.001). Strains in three of these clades (14, 16 and 18) were from 12 patients (six pairs) who had been transfused at the same shift sitting next to each other on the same bed in Amol. Two patients with strains in clade 14 and one patient with a strain in clade 16 had been infected between 1999 and 2005, while the other patient in the pairs had been infected before 1996. Clade 19 was formed by two strains from patients who had frequently been transfused at the same shift in the same room in a center in Tehran (Table 4; Figure 1c-d). One of the male patients in this clade had been infected in 2005. The 12 subtype 1b strains all diverged with more than five nucleotides from each other and did not form any clades, but were interdispersed among1b strains derived world-wide. This was significantly different as compared to the other subtypes (P < 0.001) when all strains were considered. However, most of the 1b strains, 10, were from Tehran centers, which generally had fewer similar strains circulating than the center in Amol (P = 0.015). When only the strains from Tehran centers were considered, the lack of similar 1b strains as compared to strains belonging to other subtypes was less significant (0 versus 15; P = 0.05). The patient infected with the 4a strain had been transfused for 8 years in the United Arab Emirates, and had probably been infected there.

**Table 2:** Years of Start of Transfusion and of Seroconversion to anti-HCV in 34 Thalassemia Patients Infected After 1996 in Tehran and Amol

Region	Total, No. of Patients	Cases Infected After 1996, No. (%)	Start of Transfusion		Year of Seroconversion to anti-HCV		HCV RNA Positve, No. (%)
			Before 1996, No. (%)	After1996, No. (%)	1996-2000, No. (%)	2001-2006, No. (%)	
**Tehran**	112	14 (12.5)	13 (93)	1 (7)	3 (21)	11 (79)	6 (43)
**Amol**	105	20 (19)	15 (75)	5 (25)	5 (25)	15 (75)	10 (50)
**Total**	217	34 (16)	28 (82)	6 (18)	8 (24)	26 (76)	16 (47)

**Table 3:** Results from Genotyping 130 HCV Strains from Tehran and Amol

Region	No. of genotyped strains	Genotype					
		1a, No. (%)	1b, No. (%)	2k, No. (%)	3a, No. (%)	3k, No. (%)	4a, No. (%)
**Tehran**	69	34 (49.3) a	17 (24.7) b	1 (1.4)	15 (21.8)	1 (1.4) c	1 (1.4)
**Amol**	61	35 (57.4) c	2 (3.3)	0	24 (39.3) d	0	0
**Total**	130	69 (53 )	19 (14.6)	1 (0.8)	39 (30)	1 (0.8 )	1 (0.8 )

Number of strains typed only by sequencing of the core region:

^a^4 strains;

^b^7 strains;

^c^1 strain;

^d^2 strains

**Table 4:** Strain Designations and Characteristics of Patients Infected with Related 1a and 3a HCV

Clade	Strain	Nucleotide difference	Age/sex	Year start transfusion/Year first anti-HCV positive	Origin	Clade	Strain	Nucleotide difference	Age/Sex	Year Start trans fusion/ year first anti-HCV positive	Origin
1	1072	4	30/F	1984 c	Amol	11	794 a	0	30/M	1976 c	Tehran
	1001		27/M	1981 c			752		40/M	1966/1999	
2	850	4	20/M	1988 c	Tehran	12	1053 b	5	17/M1	1990/2003	Amol
	785		27/M	1980 c			1000		9/M	1988 c	
3	1019 b	3	18/M	1989 c	Tehran	13	780	1	23/M	1983	Tehran
	1089		29/M	1992 c			779		22/F	1984 c	
	786	4-5	22/M	1985 c	Amol		1028 b	0	19/F	1987 c	Amol
	1036	4-5	12/M	2000/2001	Amol		1083		21/F	1989 c	
4	854 a	1	27/M	1979 c	Tehran	14	1083 b	3	21/F	1989 c	Amol
	843		28/M	1979 c			1011		29/F	1998/1999	
5	995 b	2	22/M	1984	Amol		1067	5	18/F	1990	Amol
	1075		17/M	1993 c			1062		24/F	1983/2001	
6	1087 b	3	28/M	1980/1996	Amol	15	727	0	26/M	1980 c	Tehran
	1031		13/M	1996/1999			733		31/M	1975 c	
	996 b	2	18/M	1991 c	Amol	16	1099 b	2	26/M	1982	Amol
	1065		16/M	1991 c			1012		18/M	1991/2005	
	1091 b	2	16/F	1989 c	Amol		1003	3-5	22/M	1985	Amol
	1047		18/F	1991			1059	4-5	35/M	1973 c	
7	1050 b	0	23/F	1989 c	Amol	17	1078	5	22/M	1981 c	Amol
	1046		25/F	1982			1082		27/M	1981 c	
8	1010 b	2	27/F	1982 c	Amol	18	1034 b	0	21/F	1981 c	Amol
	1093		27/F	1984 c			1037		27/F	1986 c	
	1015	4-5	30/M	1978 c	Amol		1037 b	1	27/F	1986 c	Amol
							1060		20/F	1989 c	
	994	3-4	13/M	1994/2002	Amol						
							1088 b		29/M	1981 c	Amol
	1025	2-5	25/M	1983 c	Amol		1090		25/M	1983 c	
9	833 a	4	22/F	1988 c	Tehran	19	746 a	5	24/F	1990 c	Tehran
	777		21/F	1988 c			871		30/F	1979/2005	
10	1066	4	21/F	1986 c	Amol						
	1048		29/F	1985 c							

^a^The patients frequently transfused at the same shift in the same room

^b^The patients were frequently simultaneously transfused while sitting next to each other on the same bed at the same shift.

^c^The patient was hepatitis C infected before 1996.

## 5. Discussion

The frequencies of subtype 1b and 3a in hepatitis C infected thalassemia patients from Tehran were 25% and 22%, respectively, which were in contrast with the findings from the thalassemia centers in Amol, where 1a and 3a were the most prevalent subtypes. In addition, data from earlier Iranian studies showed subtypes 1a and 3a were the most common ones in the country with geographical differences in their distribution, and 1a was frequent among patients infected through blood transfusion (9-11). Several factors may be responsible for the introduction of various subtypes and strains that may infect patients at the thalassemia centers in Tehran. First, 25% of the hepatitis C infected patients had been treated in other cities or abroad for several years before starting treatment in Tehran, such as the patient infected with 4a, who had been treated in the United Arab Emirates (12, 13). Second, due to a high demand for blood in Tehran, it is unavoidable for the centers to use blood supplied from other Iranian cities, which might result the introduction of various HCV subtypes. Third, sampling in Tehran was done at several thalassemia centers which may differ with regard to the number of patients, amount of blood provided from the other parts of Iran, rate of HCV infections and number of patients previously transfused in other cities or abroad. The number of new HCV infected patients decreased when blood screening for anti-HCV was introduced in Iran in 1996, the current study also indicated that 86 % of the patients had been infected before 1996. However, 34 (16 %) of the patients had become hepatitis C infected after 1996. Most of them had been infected in 2001 or later, and only eight had been infected between 1996 and 2000. This might have been an under-estimate, since there was no testing for anti-HCV in thalassemia centers on a regular basis these years. Most patients from Tehran have been tested for anti-HCV since 1998, while there was no routinely anti HCV testing in Amol until 1999 or 2000. Phylogenetic analysis of the NS5B region revealed a high genetic variability of the strains at different centers. Although 54 % of the 1a and 3a strains formed clades of similar strains, the strains from one clade were highly divergent from strains of the same subtype forming another clade. The variability between the strains and the clades may be due to several factors including transfusion of unscreened blood before 1996, blood obtained from other cities particularly for patients from Tehran, and the fact that 24% of the thalassemia patients had started their treatment in other cities. All these circumstances could lead to multiple entries of HCV lineages into the centers. However, in different centers there were closely related strains forming clades. Further epidemiological investigations revealed that 36 of these strains were isolated from patients frequently transfused at the same shift in the same room. Moreover, the number of related 1a and 3a strains from Amol was higher and these strains formed larger clades than the ones from Tehran. In addition, 71% of the patients infected with similar strains from Amol had been transfused while sitting next to each other on the same bed. This indicates that nosocomial transmission was more common in Amol thalassemia center than in those of Tehran. This could be due to Amol Thalassemia center was the only center in the city, and the number of patients often exceeded the number of beds, which could influence the patient-to-patient transmission of hepatitis C. The circulation of few similar strains, as in Amol, may also facilitate the identification of nosocomial transmission. The patients in Tehran were transfused at several centers, and could have been exposed to more divergent HCV strains. Some of the patients were also transfused and infected abroad, as the patient infected with 4a and probably the majority of those infected with 1b strains. These 1b strains were less related to each other and more similar to the strains from Western Europe (14), while the 1a and 3a strains were more similar to strains from other Iranian high risk groups. This suggests multiple entries of 1b strains from abroad into the thalassemia centers in Tehran. The high similarity of the majority of the 1a and 3a strains suggests nosocomial transmission of HCV at the thalassemia centers. In a study on five new hepatitis C cases infected at Zafar adult thalassemia clinic in 2004, all the blood donors whom could be traced, 69 %, were anti-HCV negative indicating patient-to patient transmission, although it was not shown by sequencing and genetic analysis of the patient strains (15). Nosocomial transmission of hepatitis C has previously been described between patients and at different hospital settings outside Iran (8, 16-21). However, phylogenetic analysis of the HCV strains was performed only in few of the studies to identify common source of infection or route of transmission (8, 18, 19). Until now, nosocomial HCV transmission based on sequencing and phylogenetic analysis of patient HCV strains at thalassemia centers has not previously been reported. The genetic analysis of the infecting strains in combination with further epidemiological analysis clearly indicated patient-to patient transmission of hepatitis C at the centers under study. More transmission events may have occurred than those identified, since some patients may have moved to other thalassemia centers or have died. Also, if the transmission occurred a long time ago, the strains may have diverged beyond a detectable association of the strains due to genetic drift. The exact mode of infection was not investigated, although the most likely factors may be the use of contaminated medical equipment or other poor enforcement of safety guidelines. Violation of safety procedures resulting in the spread of hepatitis C in hospitals has previously been documented (16, 22). The present study supports that nosocomial transmission plays a possible role in HCV spread at Iranian thalassemia centers. Education of staff and implementation of strict infection control practices are thus necessary in order to reduce the number of new cases of hepatitis C at these centers.
